# MVI-TR: A Transformer-Based Deep Learning Model with Contrast-Enhanced CT for Preoperative Prediction of Microvascular Invasion in Hepatocellular Carcinoma

**DOI:** 10.3390/cancers15051538

**Published:** 2023-02-28

**Authors:** Linping Cao, Qing Wang, Jiawei Hong, Yuzhe Han, Weichen Zhang, Xun Zhong, Yongqian Che, Yaqi Ma, Keyi Du, Dongyan Wu, Tianxiao Pang, Jian Wu, Kewei Liang

**Affiliations:** 1 Division of Hepatobiliary and Pancreatic Surgery, Department of Surgery, First Affiliated Hospital, School of Medicine, Zhejiang University, Hangzhou 310003, China; 2 Key Laboratory of Combined Multi-Organ Transplantation, Ministry of Public Health, Hangzhou 310003, China; 3 School of Mathematical Sciences, Zhejiang University, Hangzhou 310058, China; 4 Department of Pathology, First Affiliated Hospital, School of Medicine, Zhejiang University, Hangzhou 310003, China

**Keywords:** microvascular invasion, transformer model, contrast-enhanced computed tomography, deep learning

## Abstract

**Simple Summary:**

For early-stage hepatocellular carcinoma (HCC) (size ≤ 5 cm), the prediction of microvascular invasion (MVI) before operation is important for the therapeutic strategy. This study aimed to construct deep learning (DL) models based only on the venous phase (VP) of contrast-enhanced computed tomography (CECT), and to evaluate the performance of these models for preoperative prediction of MVI. A novel transformer-based end-to-end DL model is proposed for the first time, named MVI-TR, to capture features automatically from radiomics and to perform MVI preoperative assessments. For patient cohorts, it achieved superior outcomes in six performance measures of MVI predication status: accuracy, precision, receiver operating characteristic (ROC), area under the curve (AUC), recalling rate, and F1-score.

**Abstract:**

In this study, we considered preoperative prediction of microvascular invasion (MVI) status with deep learning (DL) models for patients with early-stage hepatocellular carcinoma (HCC) (tumor size ≤ 5 cm). Two types of DL models based only on venous phase (VP) of contrast-enhanced computed tomography (CECT) were constructed and validated. From our hospital (First Affiliated Hospital of Zhejiang University, Zhejiang, P.R. China), 559 patients, who had histopathological confirmed MVI status, participated in this study. All preoperative CECT were collected, and the patients were randomly divided into training and validation cohorts at a ratio of 4:1. We proposed a novel transformer-based end-to-end DL model, named MVI-TR, which is a supervised learning method. MVI-TR can capture features automatically from radiomics and perform MVI preoperative assessments. In addition, a popular self-supervised learning method, the contrastive learning model, and the widely used residual networks (ResNets family) were constructed for fair comparisons. With an accuracy of 99.1%, a precision of 99.3%, an area under the curve (AUC) of 0.98, a recalling rate of 98.8%, and an F1-score of 99.1% in the training cohort, MVI-TR achieved superior outcomes. Additionally, the validation cohort’s MVI status prediction had the best accuracy (97.2%), precision (97.3%), AUC (0.935), recalling rate (93.1%), and F1-score (95.2%). MVI-TR outperformed other models for predicting MVI status, and showed great preoperative predictive value for early-stage HCC patients.

## 1. Introduction

The most typical primary liver malignant tumor, hepatocellular carcinoma (HCC), poses a major risk to public health [1]. In 2020, it became the second leading cause of death for men and ranks second among all causes of cancer death worldwide [2]. With the development of technology and the change of clinical perception, liver transplantation (LT), surgical resection, targeted therapy, transcatheter arterial chemoembolization (TACE), and immune checkpoint inhibitors are the main therapeutic methods for HCC [3,4]. However, about 60% of patients will have postoperative tumor recurrence [5]. For the systematic treatment of HCC, identifying patients at high risk of recurrence is significant.

Microvascular invasion (MVI) is common in advanced HCC [6]. Different from macrovascular infiltration in advanced HCC, it is a distinct and significant predictor of recurrence after surgical treatment in early-stage HCC patients [7,8,9]. Presently, the pathological evaluation of the surgical specimens obtained after resection or LT can confirm the diagnosis of MVI, and a large tumor diameter is a prognostic factor for MVI [10,11]. An innovative tool with noninvasive and efficient recognition of MVI during early-stage HCC (tumor size ≤ 5 cm) has specific clinical importance for the determination of an individual’s treatment strategy and correspondingly the assessment of recurrence risk.

Medical imageology has been the most important method to diagnose HCC without pathological evidence [12,13]. However, the traditional clinical-radiological model lacks the indicators to predict the clinical prognosis factors, such as MVI and recurrent tumor heterogeneity [14]. With the rapid development of computer-aided diagnosis (CAD), radiomics, which can transform the original image into a great number of statistical features and explain the instinctive pathophysiology of tumors, has become one of the significant means to study the heterogeneity of many different tumors [13,15,16,17,18,19]. On the other hand, DL has achieved great success in many fields. Progressively, there has been some research in applying DL-based models to MVI histopathologic outcome prediction. These include DL-based models for MVI examining based on the images of histopathological sections [20,21]. Meanwhile, Wang and Deng [22,23] propose multimodal deep learning models based on CECT and multi-parameter magnetic resonance imaging (MRI). Based solely on CT data, Wang et al. [24] developed a light-weight transformer model for cancer segmentation and prediction. However, their validation was based on a limited number of patients and showed an unconvincing result. Yang et al. [25] applied six pretrained convolutional neural network (CNN) algorithms (pretrained using ImageNet database) to extract DL features from the sequences of non-contrast (NC), arterial phase (AP), and VP. These sequences provided more valuable information than a single image. Nevertheless, radiomics research addressing MVI diagnosis has shown a number of limitations, especially regarding prediction accuracy and generalizability. Heterogeneity exists in the source of the CNN base pretrained from the MVI.

In this study, we collected a sufficient number of large datasets of annotated medical images. These datasets were able to represent excellent universality and clinical practicality. For early-stage HCC patients (an isolated tumor size ≤ 5 cm), we proposed MVI-TR, a novel transformer-based end-to-end DL model, and developed additional DL models of MVI prediction based only on a single VP of CECT, without any clinical data. To our knowledge, we have established the first transformer-based prediction model and achieved superior accuracy and precision for MVI prediction.

## 2. Materials and Methods

### 2.1. Patients

The approval of this work was granted by the review board of our institution (the First Affiliated Hospital, College of Medicine, Zhejiang University, Hangzhou, China), and the written informed consent was waived. The Declaration of Helsinki was followed in all procedures. We collected data from 559 consecutive HCC patients, from January 2019 to December 2020, who underwent therapeutic resection or LT in our center.

The following were the inclusion criteria:

(a). Pathologically confirmed HCC after R0 resection or LT;
(b). Single tumor without satellite nodules and a lesion diameter ≤ 5 cm;
(c). Available for the pathological assessment of MVI;
(d). Receipt of preoperative hepatic CECT scan < 1 month;
(e). With well-preserved clinical and imaging information for reevaluation.

The following were the exclusion criteria.

(a). Lack of hepatic CECT within 1 month before resection or LT;
(b). Patients with recurrent HCC;
(c). Presence of extrahepatic metastases or macrovascular invasion;
(d). With multiple liver tumors;
(e). Clinical or pathological information was not available;
(f). With poor imaging quality that did not match the region of interest (ROI) definition;
(g). Patient received cancer-related preoperative treatments, including TACE, radiofrequency ablation (RFA), chemotherapy, targeted therapy, immunotherapy, or other antitumor treatments.

All patients were randomly allocated to either the training cohort (*n* = 448, MVI positive 120) or the validation cohort (*n* = 111, MVI positive 29) at a rough ratio of 4:1.

### 2.2. Clinical Characteristics and Pathological Examination

Baseline clinical information was collected for each patient, including patients’ age, sex, maximum diameter of tumors, HBV-DNA, hepatitis B surface antigen (HBsAg), and tumor markers (serum alpha-fetoprotein (AFP), carbohydrate antigen 19-9 (CA19-9), carcinoembryonic antigen (CEA), protein induced by vitamin K absence or antagonist-II (PIVKA II), etc.).

Information about pathology type and MVI was obtained under the microscope. Diagnosis of MVI was based on the HCC diagnosis and treatment guide [26]. MVI hazard levels consisted of M0 (no MVI), M1 (low risk, no more than 5 MVI inside 1 cm of tumor or adjacent to tumor), and M2 (high risk, greater than 5 MVI or MVI in non-tumor adjacent tissues). MVI negative was defined as M0, and MVI positive was described as including M1 and M2.

### 2.3. CT Data Collection

The preoperative CECT of HCC patients was retrieved from the system of picture archiving and communication in our hospital. All data were carried out on a 256-slice scanner (Brilliance iCT; Philips, Amsterdam, The Netherlands). The reconstruction of CECT images adopted the standard kernel, which has a slice thickness of 1.0 mm and an interval of 1.0 mm.

We used an automatic pump syringe to inject iodinated contrast medium and saline. CT scans were carried out at 25–35 s (AP), 50–70 s (portal vein phase), and 120–160 s (VP) after contrast injection.

### 2.4. Tumor Segmentation

The VP of CECT demonstrated better visibility of the target tumor lesion and was therefore chosen to quantify cancer features in this study. Window Width (WW) was set to 400 and Window Level (WL) was 40 to obtain proper and clear images for the surgeon [27]. The tumor segmentation on CT data was manually implemented by three hepatobiliary surgeons working independently (Cao Linping, Zhang Weicheng, and Zhong Xun) (all of them had eight years of HCC diagnosis experience) with ITK-SNAP software (3.8.0) [28], and the results were reviewed by a senior hepatobiliary surgeon (Wu Jian) with twenty years of HCC diagnosis experience. For the purpose of extracting radiomics features, we focused on the whole region of the primary lesion and contoured the domain covering it in the slice with the maximum tumor cross-sectional area.

### 2.5. Data Preprocessing

CT scans with Hounsfield units’ raw intensities were stored in Data Interchange Standard for Biomedical Imaging (DICOM) format. In order to fully utilize the information contained in the tumor region, we selected the 2D slice with the maximum tumor area from 3D CT images. Finally, the whole ROI image was resized to 224 × 224 pixels by bilinear interpolation to obtain more detailed information.

### 2.6. Data Augmentation

**Figure 1:**
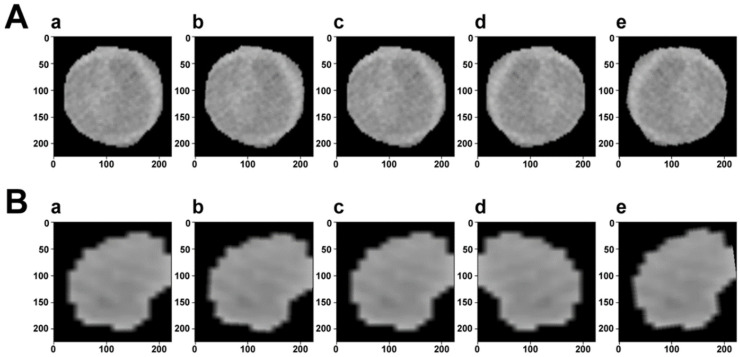
Visualization of data augmentation. The first row (**A**) is the ROI of a sample with MVI, while the second row (**B**) is without MVI. In each row, (a) is the original image. (b–d) Are generated after random rotation, random rotation crop, and random horizontal flip transformation, respectively. (e) Is the composition of the previous transformed images.

Data enhancement is a technology that enhances the quantity and quality of limited data. It can fundamentally reduce overfitting and improve the generalization of deep learning models. During the training process, we implemented different types of data enhancement, as shown in Figure 1. Because image transformations increased the diversity of data, it was conducive to overcoming problems such as scale and background, and building a more robust DL model. Experiments showed that the MVI state recognition performance of DL models had been significantly improved.

### 2.7. Deep Learning Models

There are two mainstream DL-based strategies for feature extraction, unsupervised and supervised learning methods. We first used contrastive learning for unsupervised feature extraction, which discovers hidden patterns without the need for human intervention. On the other hand, we developed different supervised learning models: MVI-TR and ResNets family. Labels are introduced in these models which further improve the performance of MVI status prediction.

#### 2.7.1. Contrastive Learning

As a self-supervised learning method, contrastive learning is effective if and only if similar instances are relatively close and dissimilar instances are farther apart in the representation space [29].

Let $f_{\theta }$ with trainable parameters θ be a backbone encoder. Referring to the work by [30], the backbone $f_{\theta }$ was pretrained on ImageNet [31]. Representation of images can be optimized independently of downstream tasks. If supervised learning is performed, the extracted information is related to the current target. We added a classifier to predict MVI status, after the pretrained backbone $f_{\theta }$.

**Figure 2:**
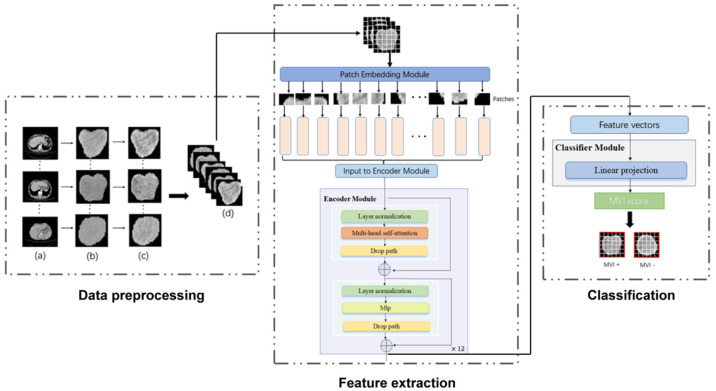
The workflow of our MVI−TR model. The entire process was divided into three parts: data preprocessing, feature extraction, and final classification. As for the data preprocessing, the region of tumor was drawn in the origin CT, as shown in (a). (b) were the cropped images that were resized into 224 × 224. (c) were the result after histogram equalization. Then (d) showed data augmentation, such as random rotation and horizontal flip. During feature extraction, a ROI was fed into the patch embedding module and then encoded in a feature vector of 768D through 12 encoder blocks. The MVI status was predicted by a linear projection on this 768D feature vector.

The framework of contrastive learning was shown in Figure 2. The backbone encoder $f_{\theta }$ extracts representation vectors from augmented data examples. A projection head of multi-layer perceptron (MLP) is applied to map representation vectors into the space of the contrastive loss. In the inference period, we threw away the projection head and used encoder $f_{\theta }$ for the downstream classification task.

#### 2.7.2. MVI-TR: A Transformer-Based Model

Transformer has been widely used in computer vision (VIT) and has achieved great success [32]. Inspired by this, we also introduced a multi-head self-attention (MSA) mechanism to extract features that reflect the relationship among different regions. As illustrated in Figure 2, MVI-TR was composed of three main components, which were stated in detail as follows:

(1) Patch Embedding module. This module divided the ROI image into a patch sequence. Then, each patch was flattened to a 1D vector by convolutional operation, and it was regarded as a token in natural language processing (NLP).

(2) Encoder module. This module consisted of 12 blocks, sharing similar structures but different parameters. We used MSA to capture the relationship between the different parts and global information. The MLP was introduced to lift to a higher dimension space and then project back to the target dimension.

(3) Classifier module. This module predicted MVI status with features extracted from the previous Encoder module.

Since parameters in different attention heads were not shared, the model was encouraged to learn the correlation among different representation subspaces, so as to better obtain the global information. As a result, the ability to predict MVI status was markedly improved.

#### 2.7.3. ResNets Family

We performed a couple of classic ResNet-like deep convolutional neural network (DCNN) architectures, ResNet18, ResNet50, and ResNet101, which are still the state-of-the-art of convolutional networks in the tasks of image classification.

ResNet18 outputs a 512D vector as features by eight convolutional blocks, and we chose a simple fully connected (FC) layer as the classifier to predict MVI status based on the features. Resnet50 and ResNet101 have the same shortcut connections as ResNet18, but with more residual blocks. For classifiers of Resnet50 and ResNet101, we adopted the convolutional and average pooling operators to derive the final probability prediction of MVI status, instead of FC layers. This further reduced the burden of computation during the inference period.

### 2.8. Regularization Techniques

Considering that our dataset was limited, we performed some effective ways to alleviate the overfitting of DL-based models:

(1) Normalization Operation. During the training period, we introduced some regularization approaches, including batch-normalization, layer-normalization, as well as a dropout for MSA, which are effective techniques to prevent deep neural networks from overfitting.

(2) Label smoothing. This technique permitted DL models to predict training samples too confidently, which was better for the improvement of generalization capability. With the noise distribution represented by u(y|x), we generated the new ground truth label as

$$p_{new}\left(y|x_{i}\right)=\left(1-\varepsilon \right)p\left(y|x_{i}\right)+\varepsilon u\left(y|x_{i}\right),$$

where *ε* ∈ (0,1) is a weighted factor, $p(y|x_{i})$ is the original one-hot encoded label.

(3) Drop path. This was an extension of dropout strategy. In MVI-TR, this approach adopted randomly dropping the operands of join layers to regularize the co-adaptation of sub-paths. Such regularization can avoid co-adaptation of paralleling paths and allow the extraction of high-performance in sub-networks with the fixed depth [33].

### 2.9. Implementation

During the training process, 448 patients were randomly selected to compose the training cohort, with 120 MVI positive patients and 328 MVI negative patients. The remaining 111 patients constituted the validation cohort, with 29 MVI positive patients and 82 MVI negative patients. When different deep learning models were trained, the same data augmentation configuration was applied on every epoch. Given that the majority of medical images had simple structure and strong similarity, we applied three main transformations, refer to Ma’s work [34], as follows:

(1) Rotation at a randomly selected angle from −10 degrees to 10 degrees;
(2) Cutting the image with a random size from 0.8 to 1.0 and a random longitudinal ratio from 0.95 to 1.05;
(3) Rolling the image horizontally with a probability of 1/2.

For supervised learning, MVI-TR and baseline ResNet-like models were all optimized through the SGD algorithm, with the same hyper-parameter configuration (a learning rate of 1/1000, a weight decay of 1/100,000, and a momentum of 0.9). We used different small batches (batch size 8, 12, and 16) to train these models and noted that, in most cases, the performance with the mini-batch of 8 was the best. The classical categorical cross-entropy loss guided the optimization of all the models. To obtain the optimal parameters, we trained over 3000 epochs on these supervised learning models. Therefore, models with higher accuracy and AUC in the validation cohort were selected for evaluation in the following parts.

## 3. Results

### 3.1. Demographic Characteristics

**Figure 3:**
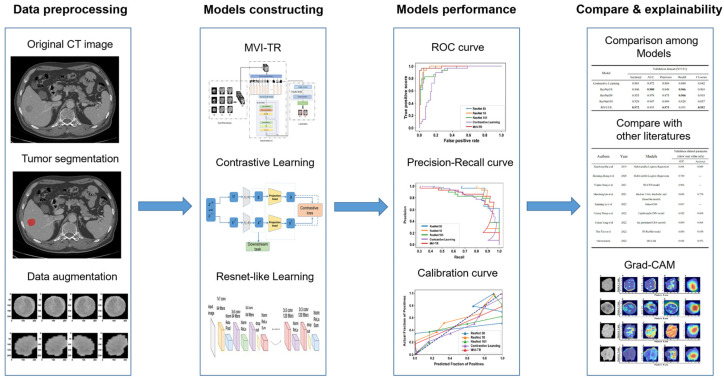
The workflow of this study. The workflow of our work included tumor segmentation, data augmentation, features extraction, model construction, and performance evaluation.

Tumor segmentation, data preprocessing and augmentation, feature extraction, and performance evaluation were all parts of the pipeline (Figure 3). In this study, 559 patients met the inclusion standard and were enrolled. They were randomly divided into the training cohort (448 patients) and the validation cohort (111 patients). The demographic characteristics of patients were compared between the training and validation cohorts (Table 1). For clinical outcomes and baseline variables, there were no significant differences between the training and validation cohorts (all *p* > 0.05).

### 3.2. Performance of MVI Prediction Models

To assess the predictive performance of the models, the following metrics were chosen: accuracy precision, AUC, receiver operating characteristic (ROC), recalling rate, and F1-score. More information is provided in Table 2.

Contrastive learning showed great performance in the training cohort, with both accuracy and AUC of nearly 1.0. However, in the validation cohort, it did not show better performance than the other methods, and only yielded an accuracy of 0.883, precision of 0.889, AUC of 0.872, recalling rate of 0.8, and F1-score of 0.842. A possible reason is that AUC is sensitive to the relative relationship between positive and negative classes. It was difficult for contrastive learning to predict whether MVI existed or not, which indicates that the contrastive learning model lacked confidence.

For supervised deep learning models, the ResNet18 model archived the highest score of AUC (0.997) in the training cohort, but its accuracy was lower than ResNet50 and MVI-TR. Additionally, although ResNet50 and ResNet101 attained better performance in the training dataset, they had lower precision in the validation dataset. This suggests that they might be worse in generalization and suffered from overfitting. Moreover, MVI-TR attained the highest accuracy (0.972), precision (0.973), and F1-score (0.952) in the validation cohort, with improved generalizability and robustness by the introduction of MSA and MLP. It outperformed other state-of-the-art methods with the highest accuracy and AUC. Further details are discussed in Section 4.

**Figure 4:**
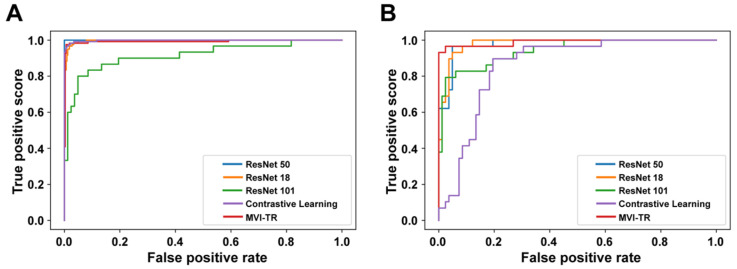
The receiver operating characteristic (ROC) curves of 5 different models. Receiver operating characteristic (ROC) curves of the contrastive learning model, MVI-TR, and baseline ResNet-like models. (**A**) ROC curves on the training cohort. (**B**) ROC curves on the validation cohort.

A receiver operating characteristic (ROC) illustrates how well a classification model performs at all classification levels. Figure 4 displays the ROC curves for the contrastive learning model, MVI-TR, and baseline ResNet-like models. The formulas for calculating the true positive rate (TPR) and the false positive rate (FPR) were Equations (1) and (2), respectively,

(1) $$TPR=\frac{TP}{TP+FN},$$

(2) $$FPR=\frac{FP}{FP+TN},$$

where TP, FN, FP, and TN denote the numbers of true positive, false negative, false positive, and true negative, respectively. To essentially show the superiority of MVI-TR, we calculated the number of model parameters and floating-point operations (FLOPs) for all models. The results showed MVI-TR with the most parameters had a large model development capability, Table 3.

### 3.3. The Advantages of MVI-TR

In this section, we provided the illustration to show that MVI-TR had the superior capability of discrimination and calibration.

#### 3.3.1. Decision Curve Analysis (DCA)

It is of great value to apply a DCA for evaluating clinical predictive models and diagnostic tests. The reason for this is that the ROC curve can only measure the diagnostic accuracy while failing to take into account the clinical utility as a specific model. The net benefit is calculated with the following equations [35]

$$\mathrm{the}\mathrm{net}\mathrm{benefit}\mathrm{treated}=\frac{TP}{N}-\frac{FP}{N}(\frac{p_{t}}{1-p_{t}}),$$

$$\mathrm{the}\mathrm{net}\mathrm{benefit}\mathrm{untreated}=\frac{TN}{N}-\frac{FN}{N}(\frac{p_{t}}{1-p_{t}}),$$

where $p_{t}$ is the threshold probability, and N is the number of samples from the validation cohort. Net benefit is the sum of the net benefit treated and the net benefit untreated [36].

**Figure 5:**
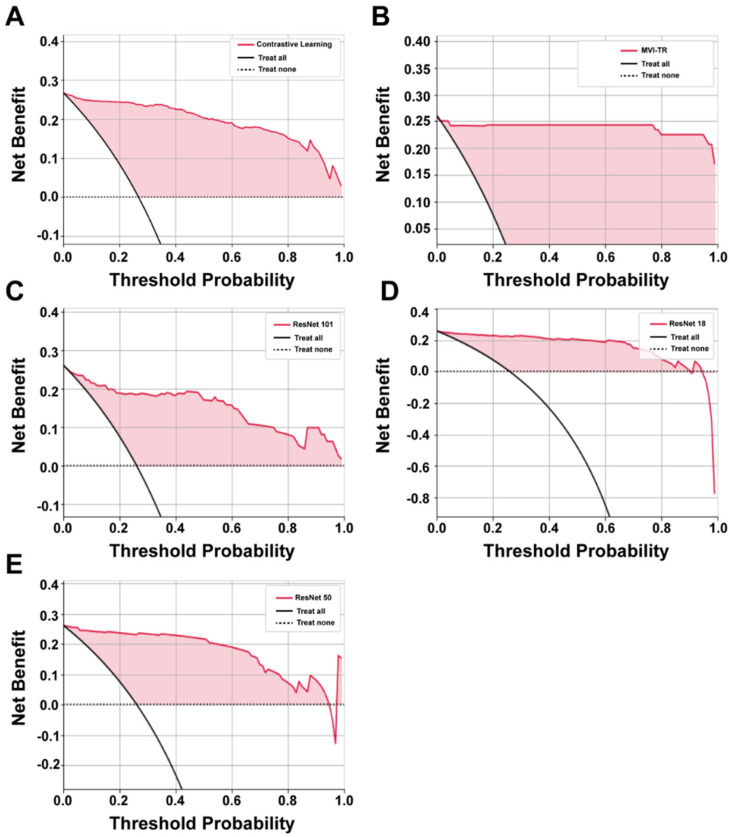
The decision curve analysis (DCA) curves of 5 different models. (**A**–**E**) Show the DCA curves of contrastive learning, MVI-TR, ResNet101, ResNet18, ResNet50, respectively.

The benefits of all the deep learning models were higher than the extreme curves, which meant they performed well in predicting MVI status, as shown in Figure 5. In particular, in a large $p_{t}$ range, the benefits of MVI-TR are much higher than the others, so its optional $p_{t}$ ranges are relatively large and safe.

#### 3.3.2. Precision-Recall (PR) Curve

**Figure 6:**
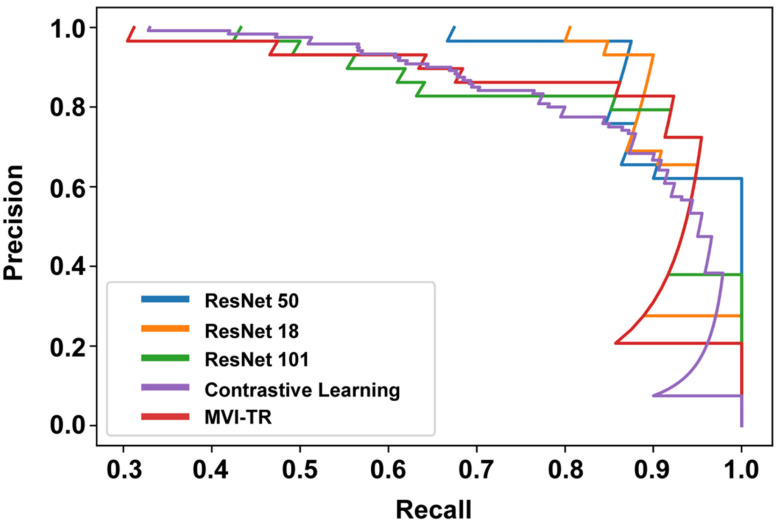
The precision-recall (PR) curves of 5 different models on the validation cohort.

The PR curve is particularly useful in reporting Information Retrieval (IR) results. IR involves searching a pool of documents to find things that are relevant to a particular user query. Different from the ROC curve, the precision-recall curve can help highlight how relevant the retrieved results are, which is more significant for accurately judging whether a patient has MVI or not. The effective algorithm should have both high precision and high recall. However, most DL-based algorithms often involve a trade-off between the two. Therefore, a favorable PR curve has a greater AUC. In Figure 6, the classification results of MVI-TR, corresponding to the red line, had a better performance.

#### 3.3.3. Calibration Curve

**Figure 7:**
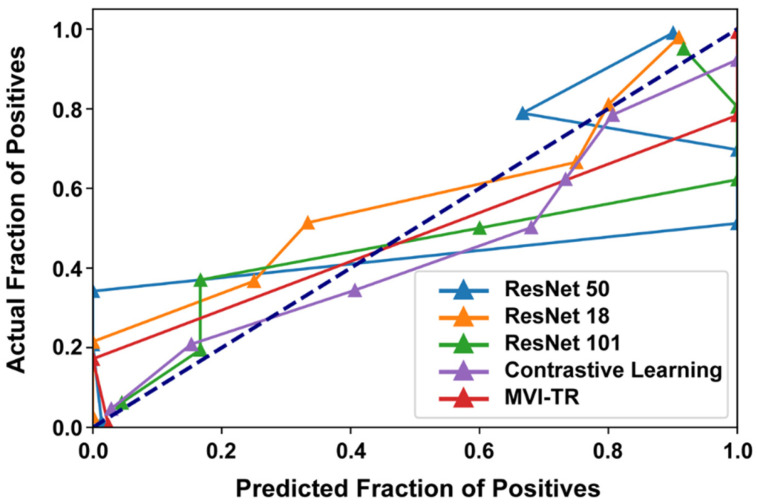
The calibration curves of 5 different models on the validation cohort.

Generally, classifiers having a linear probability of predicting the label of each class are called calibration. Therefore, a calibration curve is often used to evaluate the calibration degree of a classifier. It helps to understand the “sure” probabilities of a classification model in predicting MVI status. Additionally, it interprets how decisive a classification model is. When the calibration curve approaches the diagonal, the model achieves better performance. From Figure 7, we noted that ResNet50 and ResNet101 were not very well calibrated, tending to overfit. While MVI-TR, the contrastive learning model, and ResNet18 were closer to the perfect calibrated model’s curve (dotted curve).

### 3.4. Model Explainability

**Figure 8:**
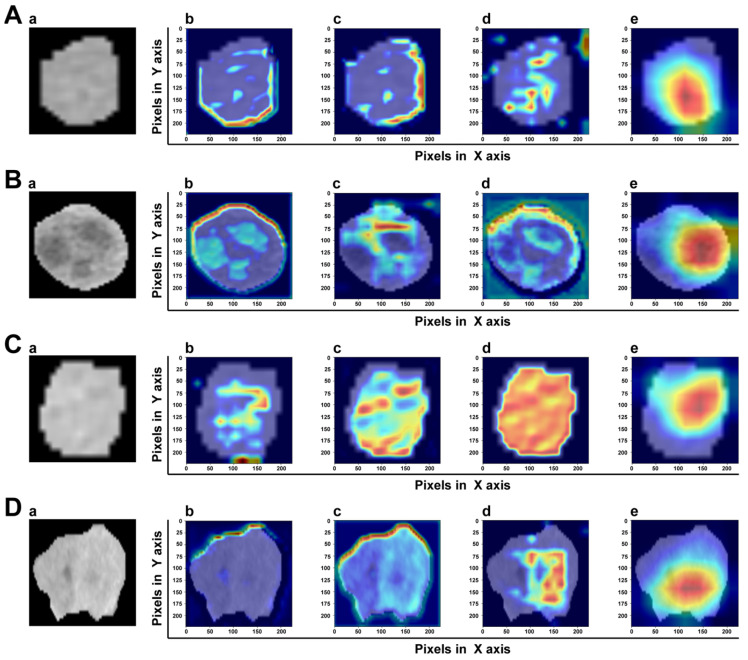
The visualization of attention which MVI-TR paid in the input ROIs by Grad-CAM. The top two rows (**A**,**B**) are the ROIs of two samples with MVI, while the bottom two rows (**C**,**D**) are without MVI. In each row, (a) is the original image. (b–d) Support for the class with MVI according to various visualizations for features of different layers in MVI-TR. (e) Is the final feature map to predict the MVI status.

A good DL model should provide high reliability and obtain the trust of physicians and patients. The gradient-weighted class activation map (Grad-CAM) can determine which essential features of an input ROI have more influence on the final prediction. In the transformer-based model, the intensities of the heatmap were set as the gradients computed by the backpropagation algorithm. Figure 8 shows the heatmap of two samples with or without MVI. It was clear that MVI-TR focused on some subdomains in the tumor areas that were highly correlated with MVI. In a sense, this provided reasonable explanations for understanding how MVI-TR performed better. It also showed that MVI-TR was unbiased, i.e., MVI-TR predicted MVI status from the information provided by the tumor, not others.

## 4. Discussion

Early-stage HCC still has a high possibility of recurrence after timely surgical resection or LT (about 50–70%) [37,38,39]. MVI and other biomarkers are reliable predictors of both overall and progression-free survival in HCC [40]. As mentioned above, the precise evaluation of MVI is very important to improve the individualized treatment strategy, reduce the recurrence for high-risk patients, and intervene in time because of the high recurrence rate after resection or LT. Partial HCC patients with MVI have benefited from TACE treatment before resection or LT [41]. Our predictive model was designed to accurately screen for MVI in HCC. As a routine preoperative method, CECT can be used as a radiological feature for MVI status prediction and MVI-related parameter generation. Our central data confirms the accuracy of the prediction models. Therefore, for patients with positive predictive outcomes, preoperative interventional therapy might be necessary to enhance clinical prognosis.

A prediction model, MVI-TR, was proposed in this exploration for predicting the absence of MVI in early-stage HCC patients (tumors size ≤ 5 cm), which showed good discrimination in both training and validation cohorts. MVI-TR of CECT data may be applied to individualized treatment guidance for HCC patients in the future.

Previous work mainly used traditional machine learning algorithms. MVI was predicted by the extraction of radiomics and the combination of clinical data. Some work adopted the LASSO algorithm to select radiomics features [34] and performed multivariable logistic regression to predict MVI status. The introduction of DL radiomics made it possible to identify MVI noninvasively and efficiently [13,25,42]. A recent study [24] combined segmentation with prediction and proposed a light-weight transformer for the segmentation and CNN for the prediction. Yang et al. [25] applied six pretrained CNN algorithms (pretrained using the ImageNet database) for the DL feature extraction on the sequences of NC, AP, and VP, which contained more valuable information than a single image. A 3D-ResNet network was also applied in MVI prediction, which improved the AUC to 0.85 [43]. Table 4 lists the performance of MVI-TR compared with previous DL work.

We introduced MSA to capture the relationship between different parts of the tumor region, which attained the best precision compared with other baseline Resnet-like models. The previous parameters were not shared while the results were finally stitched together. This allowed the model to learn relevant information in different presentation subspaces. In short, each attention head focused only on a single subspace, independent of the others. The core idea was to extract richer feature information. MSA expanded the model’s ability to focus on different locations of tumor images. Finally, by Grad-CAM, it was confirmed that MSA paid more attention to tumor regions, instead of others.

Traditional radiological features gave a better explanation for the prediction in practice and were easy to understand; however, the choosing of the features relied on the experience of radiologists. This might bring subjectivity into predictions, while MVI-TR extracted features automatically. As a consequence, the use of MVI-TR reduced bias caused by individual selection and variable factors in experience.

Our model had better robustness, which could deal with the problem of class imbalance well. We trained the model on imbalanced training data, in which there were 410 samples with MVI negative status and 149 samples with MVI positive status.

There were some limitations of this research. First, the DL-based models involved were only verified using data from a single center. The performance measurement of this model in multi-center, large cohorts, and different model scanning conditions would be of significant value in future research. Second, we chose 2D maximum tumor area for our radiomics study, which may lose information on 3D segmented tumors. Third, this study did not conduct a postoperative patient follow-up.

## 5. Conclusions

The MVI-TR derived from CECT yielded better performance for the prediction of MVI in HCC patients, compared with other DL-based models. Thus, it can help to professionally choose the optimal treatment strategies and patient management.

## Figures and Tables

**Table 1:** Demographic characteristics of patients in the training and validation cohorts.

Clinical Characteristics	All Subjects (*n* = 559)	Training Cohort (*n* = 448)	Validation Cohort (*n* = 111)	*p* Value
MVI, no (%)	559 (100)	448 (100)	111 (100)	0.888
Absence	410 (73.3)	328 (73.2)	82 (73.9)	
Presence	149 (26.7)	120 (26.8)	29 (26.1)	
Age (years), median (IQR)	58 (52–66)	58 (51.3–66)	59 (52–66)	0.835
Gender, no (%)	559 (100)	448 (100)	111 (100)	0.827
Male	444 (79.4)	355 (79.2)	89 (80.2)	
Female	115 (20.6)	93 (20.8)	22 (19.8)	
MDT (cm), median (IQR)	2.6 (2.0–3.5)	2.6 (2.0–3.5)	2.7 (2.0–3.5)	0.631
HBsAg status, no (%)	555 (99.3)	445 (99.3)	110 (99.1)	0.151
Negative	100 (17.9)	75 (16.7)	25 (22.5)	
Positive	455 (81.4)	370 (82.6)	85 (76.6)	
HBV-DNA	517 (92.5)	413 (92.2)	104 (93.7)	0.498
Detectable (≥10^3^)	154 (27.6)	126 (28.1)	28 (25.2)	
Beyond detection (<10^3^)	363 (64.9)	287 (64.1)	76 (68.5)	
AFP, no (%)	549 (98.2)	439 (98.0)	110 (99.1)	0.511
Median (IQR), ng/mL	10.6 (3.6–131.0)	11.4 (3.6–131.1)	8.5 (3.6–125.1)	
CEA, no (%)	546 (97.7)	436 (97.3)	110 (99.1)	0.499
Median (IQR), ng/mL	2.7 (1.9–4.0)	2.7 (1.8–4.0)	2.8 (2.0–4.3)	
CA19-9, no (%)	492 (88.0)	391 (87.3)	101 (91)	0.318
Median (IQR), KU/L	7.65 (4.7–14.1)	7.4 (4.7–13.7)	9.9 (5.1–15.2)	
PIVKA II, no (%)	334 (59.7)	269 (60)	65 (58.6)	0.967
Median (IQR), KU/L	82 (35–400)	83 (35–391)	71 (38.5–448)	
Therapeutic Method	559 (100)	448 (100)	111 (100)	1.000
Resection	540 (96.6)	433 (96.7)	107 (96.4)	
Transplantation	19 (3.4)	15 (3.3)	4 (3.6)	
Child Score	559 (100)	448 (100)	111 (100)	0.625
A	545 (97.5)	438 (97.8)	107 (96.4)	
B	14 (2.5)	10 (2.2)	4 (3.6)	

Note: MVI: microvascular invasion; MDT: maximum tumor diameter; AFP: alpha-fetoprotein; CEA: carcinoma embryonic antigen; CA19-9: carcinoma antigen 19-9; PIVKA II: protein induced by vitamin K absence or antagonist-II.

**Table 2:** The predictive performance of the proposed models.

Model	Training Dataset (*n* = 448)					Validation Dataset (*n* = 111)				
	Accuracy	AUC	Precision	Recall	F1-Score	Accuracy	AUC	Precision	Recall	F1-Score
Contrastive Learning	0.998	1.000	0.996	0.989	0.992	0.883	0.872	0.889	0.800	0.842
ResNet18	0.975	0.997	0.936	0.975	0.955	0.946	0.980	0.848	0.966	0.903
ResNet50	0.998	0.987	0.992	1.000	0.996	0.955	0.978	0.875	0.966	0.918
ResNet101	0.917	0.939	0.906	0.774	0.833	0.928	0.947	0.889	0.828	0.857
MVI-TR	0.991	0.980	0.993	0.988	0.991	0.972	0.935	0.973	0.931	0.952

**Table 3:** Parameters’ number and floating-point operations (FLOPs) of different deep learning models.

Model	Parameters’ Number	FLOPs
Contrastive learning	24.68 M	4132.87 M
ResNet18	11.17 M	27,335.88 M
ResNet50	35.36 M	5366.323 M
ResNet101	44.59 M	123,992.21 M
MVI-TR	85.80 M	16,863.63 M

**Table 4:** The performance of MVI-TR compared with previous deep learning work.

Authors	Year	Models	Validation Dataset Parameter (Show Max Value Only)				
			AUC	Accuracy	Precision	Recall	F1-Score
Xiaohong Ma et al. [34]	2019	Multivariable Logistic Regression	0.801	0.809	--	--	--
Xiuming Zhang et al. [44]	2020	Multivariable Logistic Regression	0.780	--	--	--	--
Yiquan Jiang et al. [45]	2021	3D-CNN model	0.906	--	--	--	0.8
Shucheng Liu et al. [42]	2021	ResNet, VGG, ResNeXt, and DenseNet models	0.845	0.770	--	--	--
Xinming Li et al. [46]	2022	DenseCNN	0.837	--	--	--	--
Liyang Wang et al. [24]	2022	Lightweight CNN model	0.922	0.868	0.875	0.827	0.8488
Yuhan Yang et al. [25]	2022	Six pretrained CNN models	0.909	0.964	--	--	--
Han Xiao et al. [43]	2022	3D-ResNet model	0.850	0.850	--	--	--
Yuhang Zhou et al. [47]	2022	Two-stage Expert-guided Diagnosis	0.766	0.572	0.676	0.517	0.807
Our research	2023	MVI-TR	0.935	0.973	0.973	0.931	0.952

## Data Availability

The data presented in this study are available on request from the corresponding author.

## Abbreviations

AFP	Alpha-fetoprotein
AP	Arterial phase
AUC	Area under the receiver operating characteristic curve
CA19-9	Carbohydrate antigen 19-9
CAD	Computer-aided diagnosis
CEA	Carcinoembryonic antigen
CECT	Contrast-enhanced Computed Tomography
CNN	Convolutional Neural Network
DCNN	Deep convolutional neural network
DL	Deep learning
FC	Fully connected
FNN	Feed-forward neural network
Grad-CAM	Gradient-weighted class activation mapping
HBsAg	Hepatitis B surface antigen
HCC	Hepatocellular cancer
LT	Liver transplantation
MLP	Multi-layer perceptron
MSA	Multi-head self-attention
MVI	Microvascular invasion
MVI-TR	Transformer-based end-to-end DL model
NC	Non-contrast
NLP	Natural language processing
PIVKA II	Protein induced by vitamin K absence/antagonist-II
ResNets	Residual networks
RFA	Radiofrequency ablation
ROI	Region of Interest
TACE	Transcatheter arterial chemoembolization
VP	Venous phase
WL	Window Level
WW	Window Width
